# Supplementary material for: Mycogenic Metal Nanoparticles for the Treatment of Mycobacterioses
Source: Antibiotics (Basel). 2020 Sep 2;9(9):569. doi: 10.3390/antibiotics9090569 (PMC7559022; doi:10.3390/antibiotics9090569)
Supplement: Supplementary file 1 [file antibiotics-09-00569-s001.pdf]

antibiotics

MDPI

Supplementary Table 1. Recent reports on mycosynthesis of AgNPs.

AgNPs size	AgNPs shape	Fungal species used on the synthesis	Highlights	Ref.
3-30 nm	Roughly spherical	*Aspergillus niger*	Colloidal, stable and polydispersed. Antifungal activity against fungi, *A. niger*, and antibacterial activity against both Gram-positive, *Staphylococcus* sp. and *Bacillus* sp., and Gram-negative *Escherichia coli*	[87]
≈15 nm	Spherical (face centered cubic)	Mycelia of *Rhizopus oryzae*	Strong activity against *E. coli* and *Staphylococcus aureus*. Stable for 3 months	[88]
8-60 nm	Spherical	*Trichoderma* spp.: *T. asperellum*, *T. harzianum*, *T. longibrachiatum*, *T. pseudokoningii*, and *T. virens*	*T. virens* produced the highest concentration of AgNPs	[89]
4-31 nm	Spherical	*Alternaria* sp., *Fusarium oxysporum*, *Curvularia* sp., *Chaetomium indicum*, and *Phoma* sp. (isolated from the leaves of ferns)	Stable and polydispersed. Antiviral capacity against herpes simplex virus, types 1 (HSV-1) and 2 (HSV-2), and human parainfluenza virus type 3 (HPIV-3), reducing viral infectivity	[90]
25-30 nm	Spherical	*Penicillium* sp. (endophytic; isolated from *Curcuma longa*)	Well dispersed. Good activity against MDR *E. coli* and *S. aureus*	[91]
NR	NR	*Fusarium semitectum*	Activity against 4 different pathogens. Best activity against Gram-positive bacteria: highest against *Streptococcus pyogenes*, followed by *S. aureus*, *Pseudomonas aeruginosa*, and *Salmonella typhi*	[92]
20-80 nm	Spherical	*Candida albicans*	Significant antimicrobial activity, which was more pronounced against *S. aureus* than *E. coli*	[93]
3-30 nm	Spherical	*Penicillium politans*	Activity against *Bacillus subtilis*, *Bacillus pumilus*, *Bacillus mycoides*, *S. aureus*, *E. coli*, and *C. albicans*	[94]
≈60 nm	Spherical	*Penicillium* spp. (isolated from the plant *Glycosis mauritiana*)	Strong antioxidant, antibacterial, anti-inflammatory, antilipoxygenase, xanthine oxidase and tyrosine inhibitory activities	[95]
≈22 nm	Spherical	Calcium alginate encapsulated biomass of *Phoma exigua* var. *exigua*	Colloidal and stable NPs. Antibacterial activity against *E. coli* and *S. aureus*	[96]
18-25 nm	Spherical	Macro- and Micro-fungi from soil: *Pleurotus ostreatus* *Lactarius glaucescens* *Trichoderma strigosellum* *Myrothecium verrucaria* *Penicillium striatisporum*	Activity against *Streptococcus pyogenes*, *S. aureus*, *Bacillus cereus*, *E. coli*, and *C. albicans* with enhanced synergistic effects with antibiotics. Highest antimicrobial potential against tested clinical pathogens found with AgNPs macrofungi.	[97]

4-16 nm	Spherical and oval	*Trichoderma viride*	Activity against *B. mycoides*, *E. coli*, and *C. albicans*. Use of statistical-aided approach (response surface methodology)	[98]
50-70 nm	Spherical	*Rhizopus arrhizus*, *Trichoderma gamsii*, and *A. niger*	Activity against all tested Gram-positive and -negative bacteria	[48]
12 nm	Spherical	*Chaetomium globosum*	Activity against *S. aureus* and *Klebsiella pneumoniae*	[99]
11-20 nm	Spherical	*Penicillium* spp.	Activity against the Gram-positive *S. aureus*	[100]
3-20	Spherical	*A. niger*	Activity against *B. mycoides*, *E. coli*, and *C. albicans* Synthesis optimization via statistical methods (central composite design)	[101]
≈20-80 nm	Spherical	*Aspergillus fumigatus*	Activity against *B. mycoides*, *E. coli*, and *C. albicans*	[102]
≈25 nm	Spherical and tetrahedron	*Talaromyces purpureogenus*	Dispersed. Activity against several pathogenic bacteria	[86]
10-35 nm	Spherical	*Tritirachium oryzae*	Polydispersed, with antimicrobial activity against drug-resistant or MDR bacteria (MIC = 6.38-19.15 µg/mL)	[103]
60-85 nm	Anisotropic	*A. niger* isolated from soil	Polydispersed, with antimicrobial activity against MDR *S. aureus* strains	[104]
4-26 nm	Spherical	*A. niger*, *A. fumigatus*, *Aspergillus flavus*, *Aspergillus nidulans*, *F. oxysporum*, *Nigrospora oryzae*, *Penicillium chrysogenum*, and *Trichoderma harzianum* (endophytes isolated from the plant *Calotropis procera*)	All AgNPs presented antibacterial activity, but those from *A. fumigatus*, *A. flavus* and *P. chrysogenum*, were the most active, with the last two also having the highest antifungal activity. *A. nidulans*, *N. oryzae* and *T. harzianum* did not present any antifungal activity	[105]
182.5 ± 6.9 nm	Spherical	*T. harzianum*	Antifungal activity	[106]
88 ± 7.3 nm	Spherical	Co-culture of *T. harzianum* and *Sclerotinia sclerotium*	Antifungal activity	[106]
10-15 nm	Spherical	Endophytic isolates of *Trichoderma atroviride*	Antibacterial and antifungal activity	[55]

MDR, multidrug resistant; MIC, minimum inhibitory concentration; NPs, nanoparticles; Ref., reference.
